# Rhabdopleurid epibionts from the Ordovician Fezouata Shale biota and the longevity of cross-phylum interactions

**DOI:** 10.1038/s42003-023-05377-x

**Published:** 2023-10-11

**Authors:** Karma Nanglu, Madeleine E. Waskom, Jared C. Richards, Javier Ortega-Hernández

**Affiliations:** Museum of Comparative Zoology and Department of Organismic and Evolutionary Biology, Harvard University, 26 Oxford Street, Cambridge, MA 02138 USA

**Keywords:** Palaeontology, Ecology

## Abstract

Evidence of interspecific interactions in the fossil record is rare but offers valuable insights into ancient ecologies. Exceptional fossiliferous sites can preserve complex ecological interactions involving non-biomineralized organisms, but most of these examples are restricted to Cambrian Lagerstätten. Here we report an exceptionally preserved cross-phylum interspecific interaction from the Tremadocian-aged Lower Fezouata Shale Formation of Morocco, which consists of the phragmocone of an orthocone cephalopod that has been extensively populated post-mortem by tubicolous epibionts. Well-preserved transverse bands in a zig-zag pattern and crenulations along the margin of the unbranched tubes indicate that they correspond to pterobranch hemichordates, with a close morphological similarity to rhabdopleurids based on the bush-like growth of the dense tubarium. The discovery of rhabdopleurid epibionts in the Fezouata Shale highlights the paucity of benthic graptolites, which also includes the rooted dendroids *Didymograptus* and *Dictyonema*, relative to the substantially more diverse and abundant planktic forms known from this biota. We propose that the rarity of Paleozoic rhabdopleurid epibionts is likely a consequence of their ecological requirement for hard substrates for initial settlement and growth. The Fezouata rhabdopleurid also reveals a 480-million-year-old association of pterobranchs as epibionts of molluscs that persist to the present day.

## Abstract (web summary)

From the Fezouta Shale of modern-day Morocco comes fossil evidence of a cross-species interaction showing tubulous epibionts having lived on the phragmocone of a dead cephalopod.

## Introduction

The Lower Paleozoic fossil record has produced substantial insights into the macroevolution and paleoecology of some of the earliest animal-dominated communities^1,2^. The preserved morphology of extinct species can be used to infer various aspects of their functional ecology, such as mode of feeding^3,4^, reproductive behavior^5,6^, and even vertical migration patterns^7^. More rarely, the fossil record can also capture direct paleoecological interactions between different types of organisms, which provide a much more nuanced view of the dynamic complexity of marine ecosystems in deep time. Relatively common types of interspecific ecological interactions include evidence of durophagy^8^, symbiosis^9^, and parasitism^10^, all of which have been reported from the conventional shelly fossil record. However, the Lower Paleozoic contains abundant sites with exceptional fossil preservation, which refines our understanding of the diversity of direct biotic interactions expressed in these early ecosystems. Cambrian Burgess Shale-type biotas have revealed striking cases of mutualism among soft-bodied vermiform organisms^11^, host-specific infestations^12^, evidence of symbiotic fouling^13^, and several peculiar cases of epibiontic behavior involving brachiopods as either hosts^14^ or commensals^15^. In some cases, the ecology of some organisms will facilitate the fossilization of biotic interactions, such as the case of epibionts that require a hard substrate to metamorphose from the larval to the adult stage, as in the case of some sessile echinoderms^16^ and benthic pterobranchs^17,18^. Although post-Cambrian Paleozoic sites also contain evidence of biotic interactions^19,20^, Burgess Shale-type preservation becomes much rarer after the Miaolingian and thus restricts access to this type of valuable ecological data.

**Fig. 1:**
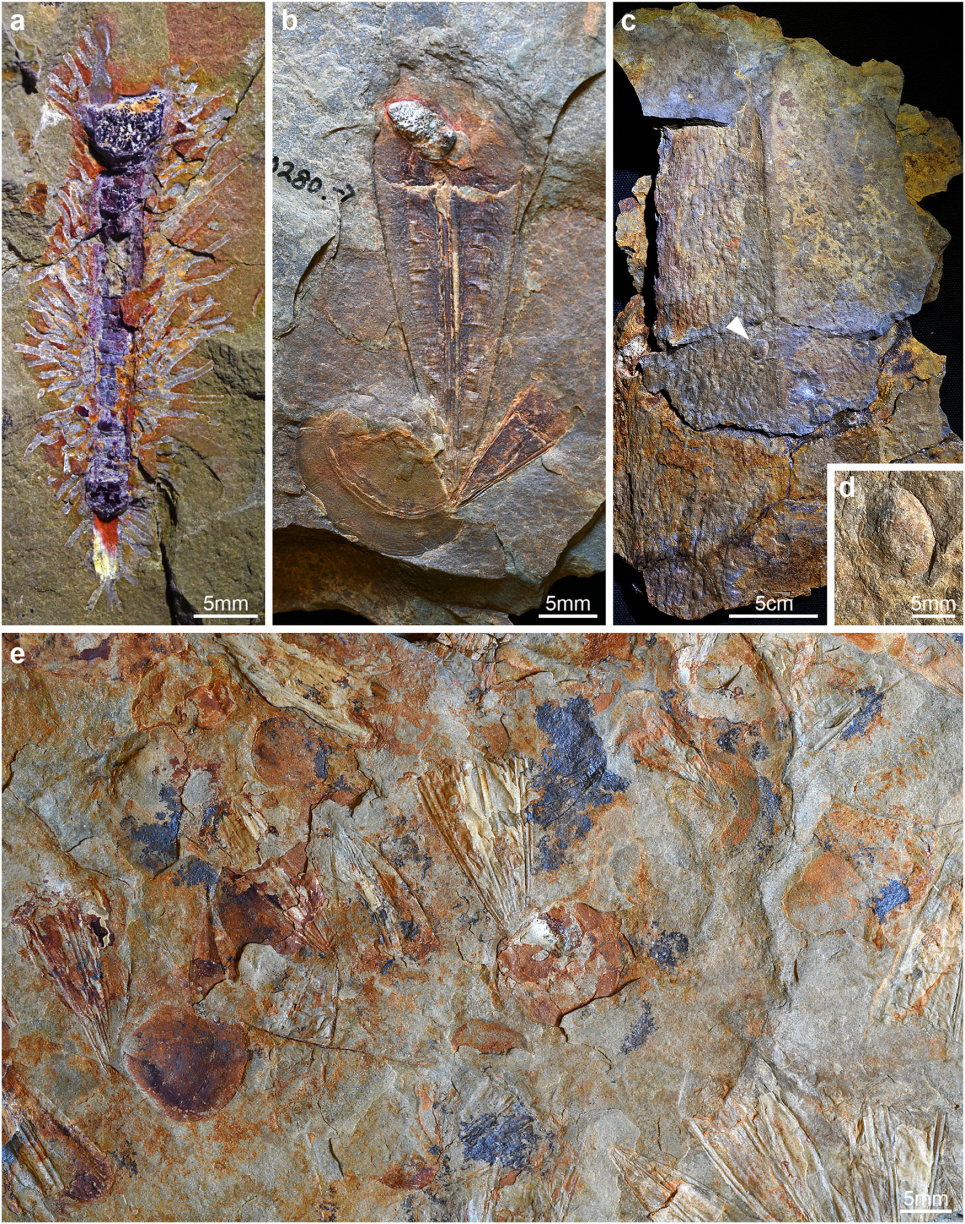
Fossil evidence of epibiontic interactions in the Ordovician Fezouata Shale biota of Morocco. **a** MCZ.IP.198903a, association of rhabdopleurid epibonts on a cephalopod. **b** YPM.IP.530280, association of two conulariids growing on a brachiopod shell. **c** MCZ.IP.198899, a large *Aegirocassis* dorsal carapace with putative brachiopod epibiont (arrowhead). **d** Close-up of brachiopod epibiont. **e** YPM.IP.524407, faunal slab with several cases of conulariids growing on brachiopod shells.

In this context, the Fezouata Shale biota from the Lower Ordovician of Morocco is of critical importance. The Fezouata Shale is an exceptional marine deposit that preserves a diverse and abundant fossil assemblage including both biomineralized and non-biomineralized organisms^21^. It was deposited under the fair-weather wave base, however, the majority of the most exceptional fossil preservation is restricted to below the storm-weather wave base, underscoring the complex depositional setting of the locality^22–25^. The biodiversity of the Fezouata Shale biota combines faunal elements from Cambrian Burgess Shale-type biotas (e.g., marrellomorphs, radiodonts; palaeoscolecids)^26–28^, alongside more “modern” groups, more typical of the Paleozoic evolutionary fauna (e.g., asterozoans, synziphosurans, machaeridians)^20,29,30^. The Tremadocian-Floian age of the Fezouata Shale biota makes it the best available view into the early Ordovician world, both in terms of its evolution and ecology. However, despite the substantial biodiversity and abundance of animal fossils preserved in the Fezouata Shale biota, including striking cases of complex collective behavior^31^, actual interspecific ecological interactions between different species have been rarely recorded (Fig. 1). For example, Van Iten et al.^32^, described cases of epibiontic attachment between brachiopods and the conulariid *Eoconularia* (Fig. 1b, d, e), while Saleh et al.^24^ have described brachiopods which may have been attached to isolated dorsal carapaces of the radiodont *Aegirocassis* (Fig. 1c, d). These examples of brachiopods attaching to other taxa, while undoubtedly important for reconstructing the ecology of the Fezouata biota, are notably common throughout the fossil record, including in sites with more conventional preservation quality (see Ager^33^ for a review). This relative paucity of data is remarkable considering the often cited similarity of faunal composition^21^ and preservational quality between Fezouata and Cambrian Lagerstätten^11–15^ (however, see Saleh et al.^25^ for a full description of the differences in soft-tissue preservation between the Walcott Quarry and Fezouata). In this context, Cambrian Lagerstätten are notable for containing a wide diversity of interspecific and cross-phylum ecological interactions^11–15^, which are comparatively uncommon at Fezouata. This contrast is even more pronounced considering that it has been proposed that the Fezouata Shale biota preserves all components of the water column equally, removing one major aspect of taphonomic bias that may normally preclude the preservation of interspecific interactions (Saleh et al.^34,35^).

Here, we report a new cross-phylum fossilized ecological association from the Fezouata Shale biota, consisting of the phragmocone of a cephalopod that has been posthumously inhabited by a dense colony of benthic pterobranch graptolites (Fig. 1a). This discovery has direct implications for our understanding of the ecology of the Fezouata Shale biota, the fossil record and evolutionary history of benthic graptolites, and the nature of early Ordovician^36^ marine ecosystems more broadly.

## Results and discussion

**Fig. 2:**
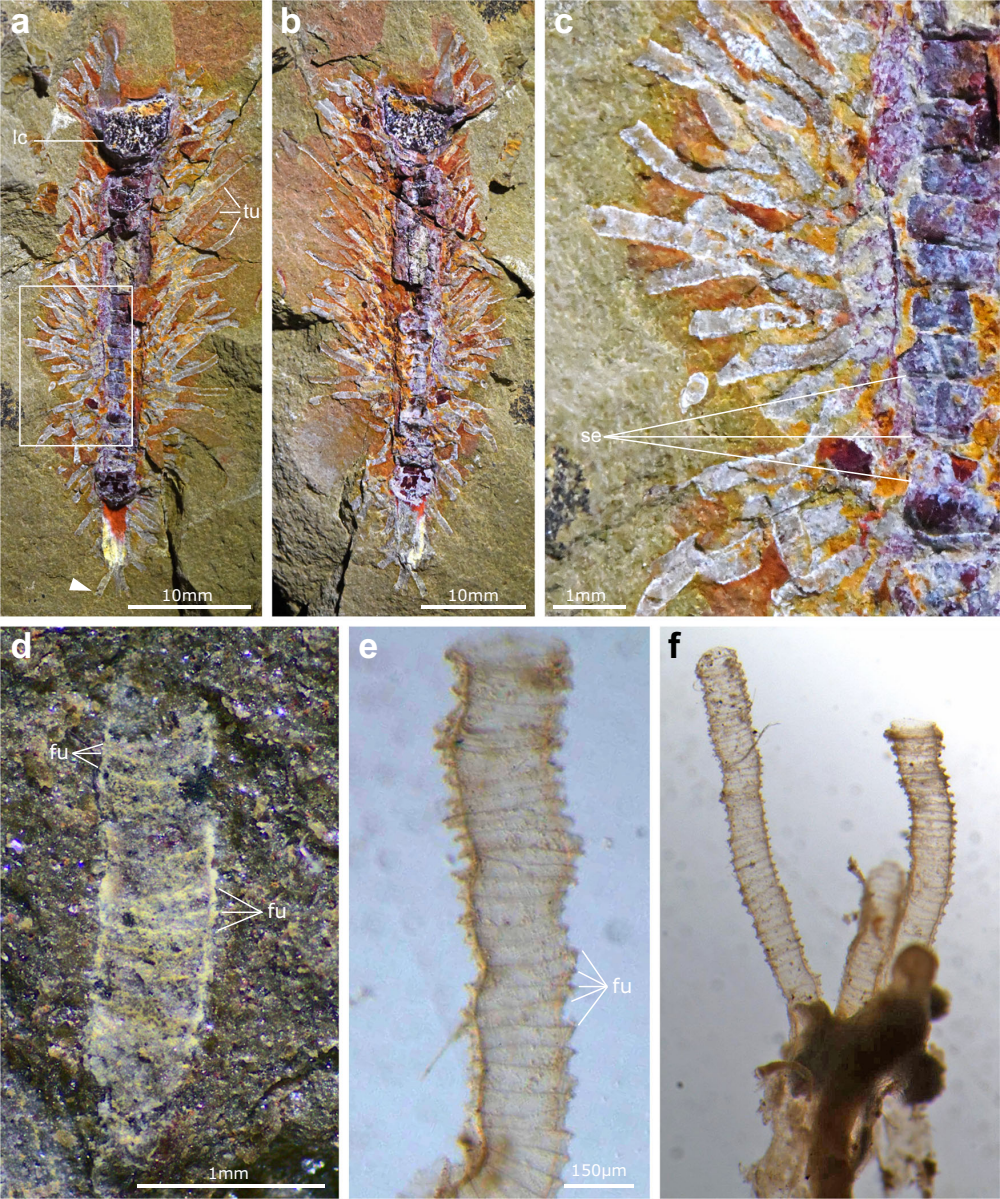
Ecological interaction of rhabdopleurid epibionts growing on a cephalopod phragmocone from the Tremadocian Lower Fezouata Shale. **a** MCZ.IP.198903a, phragmocone with densely populated tubarium of rhabdopleurid hemichordates consisting of 88 individual tubes. **b** MCZ.IP.198903b, counterpart. **c** Close-up of a dense section of the hemichordate tubarium (box in panel (**a**)), showing that individual tubes are unbranched yet overlapping. **d** Close-up of the well-preserved tube (indicated by the white arrowhead in panel (**a**)), showing the fusellar rings diagnostic of pterobranch hemichordates. **e** Transmitted light photograph of the tube of the extant hemichordate *Rhabdopleura annulata*, showing the variability of the fusellar rings as either stacked parallel bands or as a zig-zag pattern and marginal crenulations. **f** Transmitted light photograph of *R. annulata* tubes showing a similar organization as found in MCZ.IP.198903. fu: fusellar rings, lc: living chamber, se: septum, tu: pterobranch tubes.

**Fig. 3:**
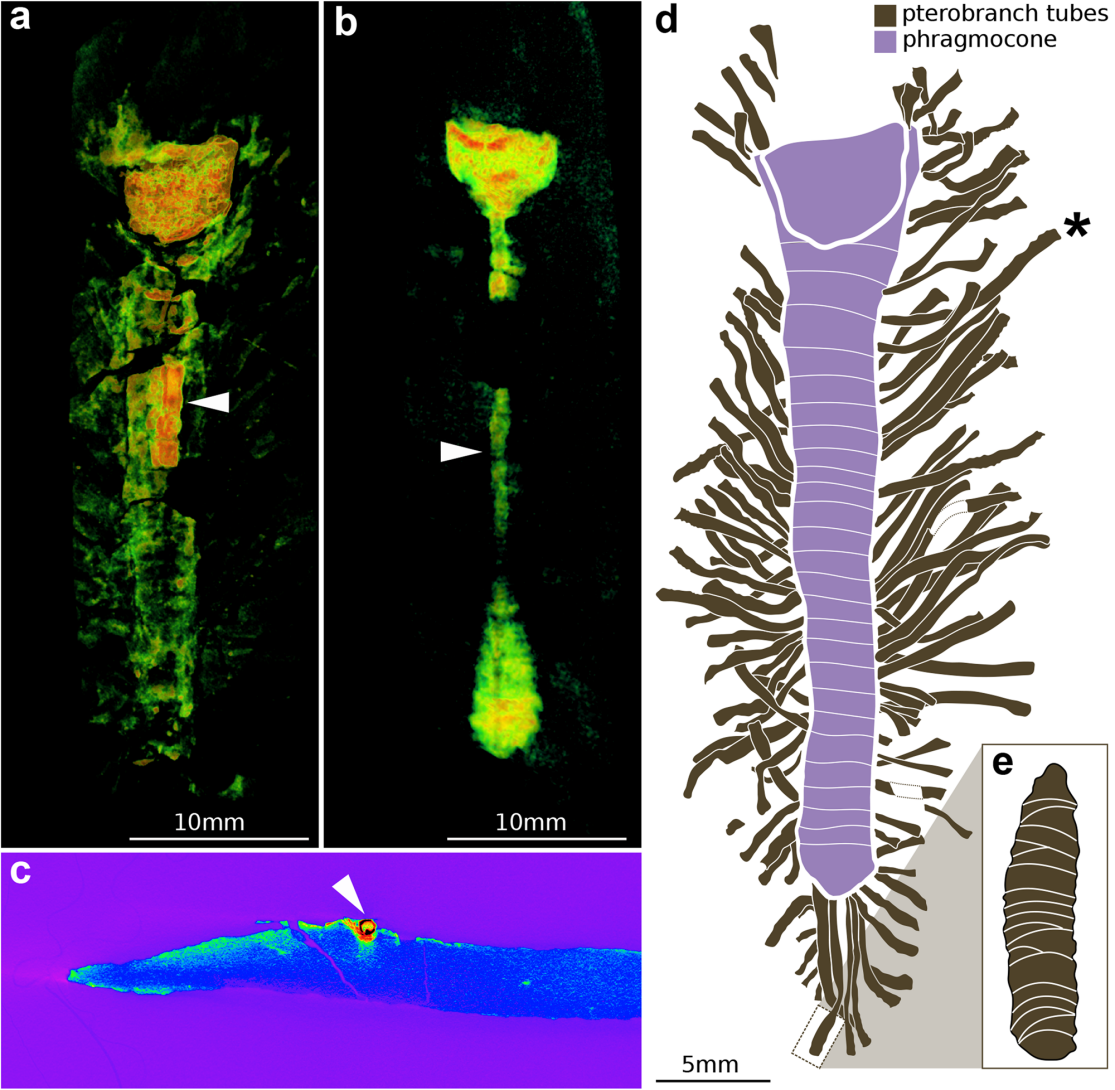
Tomographic data and interpretative diagrams of MCZ.IP.198903. **a** Tomographic three-dimensional model orthocone phragmocone. Warm colors indicate high-density areas. The septa (colored in yellow) are markedly dense, and the pterobranch tubes (colored in green) are slightly denser than the surrounding matrix. The white arrowhead indicates the siphuncle, which is of higher density than the rest of the phragmocone. **b** Tomographic three-dimensional model of the counterpart of MCZ.IP.198903. The counterpart clearly shows the siphuncle (white arrowhead) as a high-density material living chamber. **c** Transverse section of the counterpart of MCZ.IP.198903, showing that the high-density siphuncle can be discriminated from the surrounding phragmocone and less dense rock matrix. **d** Interpretative drawing of MCZ.IP.198903 illustrating the 88 pterobranch tubes; asterisk indicates longest measured tube. **e** Interpretative drawing of the best-preserved tube with fusellar rings, illustrated in Fig. 1d.

MCZ.IP.198903 represents a composite fossil preserved flattened on a siltstone matrix (Figs. 1a, 2a–c and 3a–c; Supplementary Fig. S1). The axial portion of the specimen consists of an orthocone cephalopod phragmocone with a length (sagittal) of 39.1 mm, a maximum width (transverse) of 6.1 mm at the living chamber, and a gentle 6° angle tapering to the distal point (Figs. 1a and 2a–c). The phragmocone is heavily replaced by dark reddish iron oxides and preserved with considerable convexity, with the living chamber being notably three-dimensional and featuring a granular texture. At least 25 transverse septa are clearly distinguishable throughout the length of the phragmocone (Figs. 1a, 2a–c and 3d), with each chamber also featuring three-dimensional preservation, although not to the same extent as the living chamber. Computed tomography reveals that the medially located siphuncle is well preserved (Fig. 3a–c), confirming its identification as a cephalopod phragmocone despite its modified appearance due to the proliferation of iron oxides.

The perimeter of the phragmocone is densely covered by light-colored tubes (*N* = 88) (Figs. 1a and 2a–c). The tubes have a consistent width (trans.) of approximately 0.5 mm and a variable length (sag.) ranging from 1.2 mm to 10.2 mm, with the longest tube measured from its attachment base on the phragmocone to its distal opening (Fig. 2d). Close examination indicates that the tubes consist of a single continuous element, without evidence of branching or bifurcation (Fig. 2c, d). However, there are several instances in which individual tubes closely overlap, particularly at their proximal attachment base to the phragmocone, giving the impression of a dichotomous organization (Fig. 2c). Despite this overlap, we find no evidence of true branching on any of the 88 tubes preserved in MCZ.IP.198903 (Fig. 3c, d). There is no indication of soft tissues or internal organs preserved within the walls of any of the individual tubes in MCZ.IP.198903.

Despite their small size, the tubes are exceptionally well preserved and contain fine morphological details that inform their construction and phylogenetic affinities (Fig. 2c, d). The tubes possess a distinctive banding pattern consisting of zig-zag-shaped transverse septa throughout their entire length, coupled with finely crenulate margins at either side of the tube walls (Fig. 2c, d). The distinctive pattern indicates that the tubes were secreted by pterobranch hemichordates^37^ (Fig. 2e, f). In this context, the bandings would correspond to fuselli, which are produced through the characteristic growth mode of pterobranchs and, by extension, graptolites which are now regarded as derived members of this group^38^. The fusellar organization observed in MCZ.IP.198903 is nigh-identical to that of the genus *Rhabdopleura*^39^ (Fig. 2e, f), which have been recently recognized as the only living representatives of the graptolite lineage^38^. In both MCZ.IP.198903 and extant *Rhabdopleura*, individual bands may appear to be roughly parallel to each other, or they may form a zipper-like arrangement where one band curves into another (Figs. 2d–f and 3e). Although it is not possible to obtain elemental data on the tubes due to the size of the slab containing MCZ.IP.198903, their interpretation as pterobranchs implies that they most likely have a collagenous composition^37^.

The precise organization of the tubes that form the colony in extant pterobranchs (i.e., the tubarium) has direct implications for their classification. In the case of MCZ.IP.198903, the tubarium is organized like a bush, with individual tubes extending directly adjacent to each other from the phragmocone. This organization and the style of fusellar bands indicate that this represents a thigmophilic rhabdopleurid, as opposed to a runner-type rhabdopleurid with less dense colonies^37^. Although there is no direct fossil evidence showing the soft-tissue connection between individual tubes nor the remnants of the stolon system, we regard the fossil as representing a fully basally connected colony by comparison with the habitus observed in extant rhabdopleurids in which the periderm covers the hard substrate completely^37^.

### Taphonomic interpretation and ecological significance of MCZ.IP.198903

**Fig. 4:**
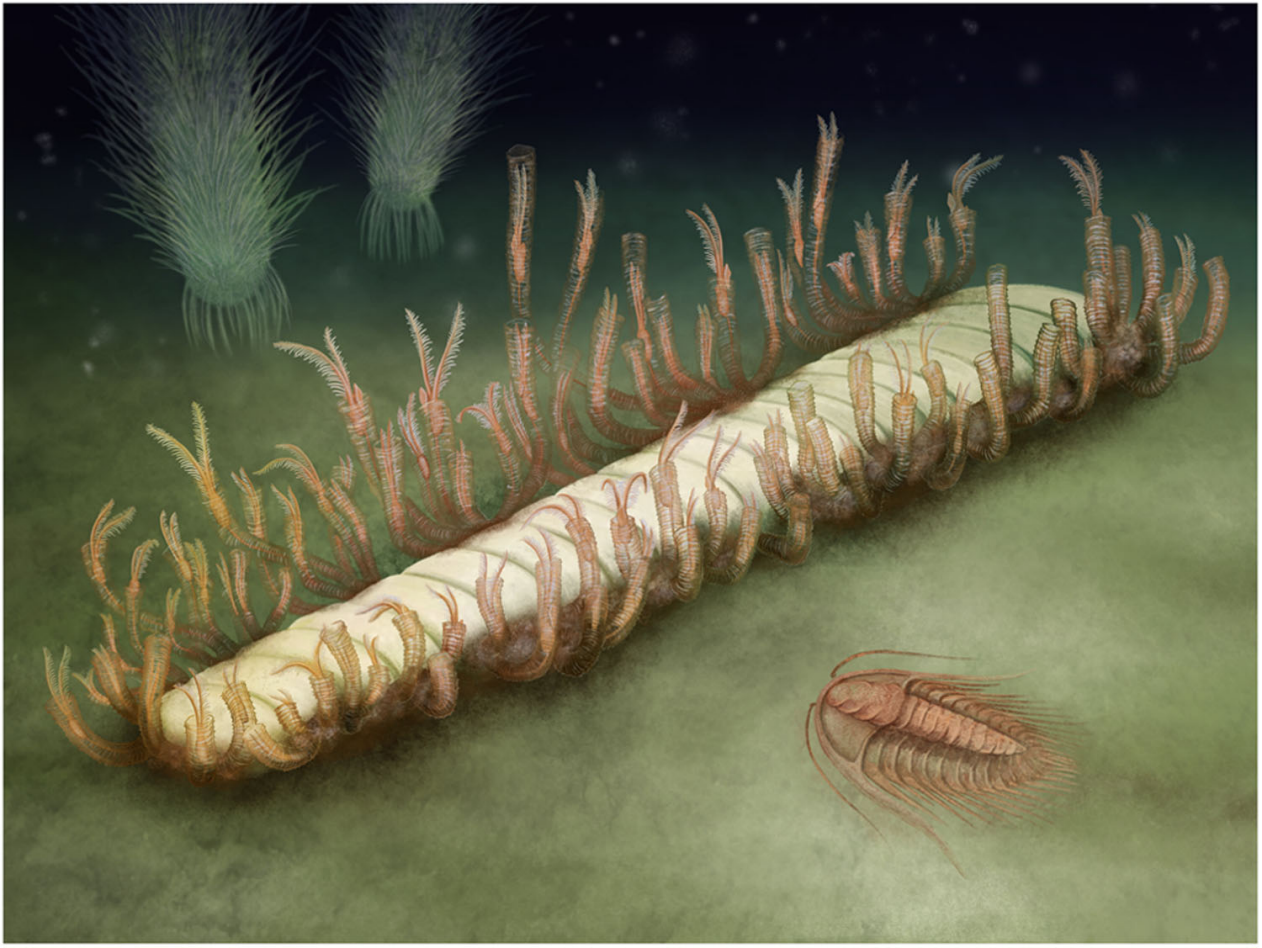
Environmental reconstruction of rhabdopleurid epibionts from the Fezouata Shale biota growing on cephalopod phragmocone. Zooids in their tubes are based on the extant genus *Rhabdopleura*. Artwork by Franz Anthony (https://franzanth.com/).

The presence of 88 well-preserved individual tubes of various lengths associated with the exposed phragmocone leads us to interpret MCZ.IP.198903 as a case of post-mortem colonization of the orthocone by rhabdopleurid-like pterobranch epibionts based on several lines of evidence (Fig. 4). First, through comparisons with the growth rate extant *Rhabdopleura*^40^, we estimate a minimum age for the formation of the pterobranch colony in MCZ.IP.198903 of approximately 28 days, calculated from measuring the size of the best-preserved fuselli (Fig. 2d; average length between fuselli = 0.1366 mm; standard deviation = 0.0524 mm) and extrapolated to the longest tubes preserved in the specimen (length = 10.2 mm; Fig. 2d); this observation allows us to establish a minimum timeframe for the development of the colony once settled on the hard substrate. Second, decay experiments show that *Rhabdopleura* zooids will degrade completely within a few days but that the collagenous tubes can maintain their overall integrity for at least 10 weeks under anoxic conditions^41,42^; this comparison suggests that the pterobranch colony in MCZ.IP.198903 most likely persisted on the seafloor undisturbed for a few weeks after death, possibly caused by an abrupt change of the local environment given the lack of signs of predation or bioturbation. Third, MCZ.IP.198903 clearly shows that the outer layers of the orthocone shell are absent, revealing the internal chambers of the phragmocone replicated in three-dimensional iron oxides; this observation indicates that the cephalopod shell underwent substantial decay and an advanced degree of diagenetic alteration on the seafloor. Taken together, we argue that the cephalopod was already a carcass for some time, enough to cause the loss of the outer layers of the shell, and subsequently overgrown by the rhabdopleurid epibionts during a minimum period of one month prior to death and subsequent burial (Fig. 4). This observation also agrees with sedimentological data suggesting that the vast majority of the best-preserved material from the Fezouata Shale was located below the storm-wave base^23,25^. Only large storms would have the energy necessary to transport sediments to this type of depositional setting. In this context, it is unsurprising that the dead cephalopod carcass lay open to ambient conditions long enough for all soft tissues to decay, larval hemichordates to establish, and then grow to a colony of 88 zooids.

**Fig. 5:**
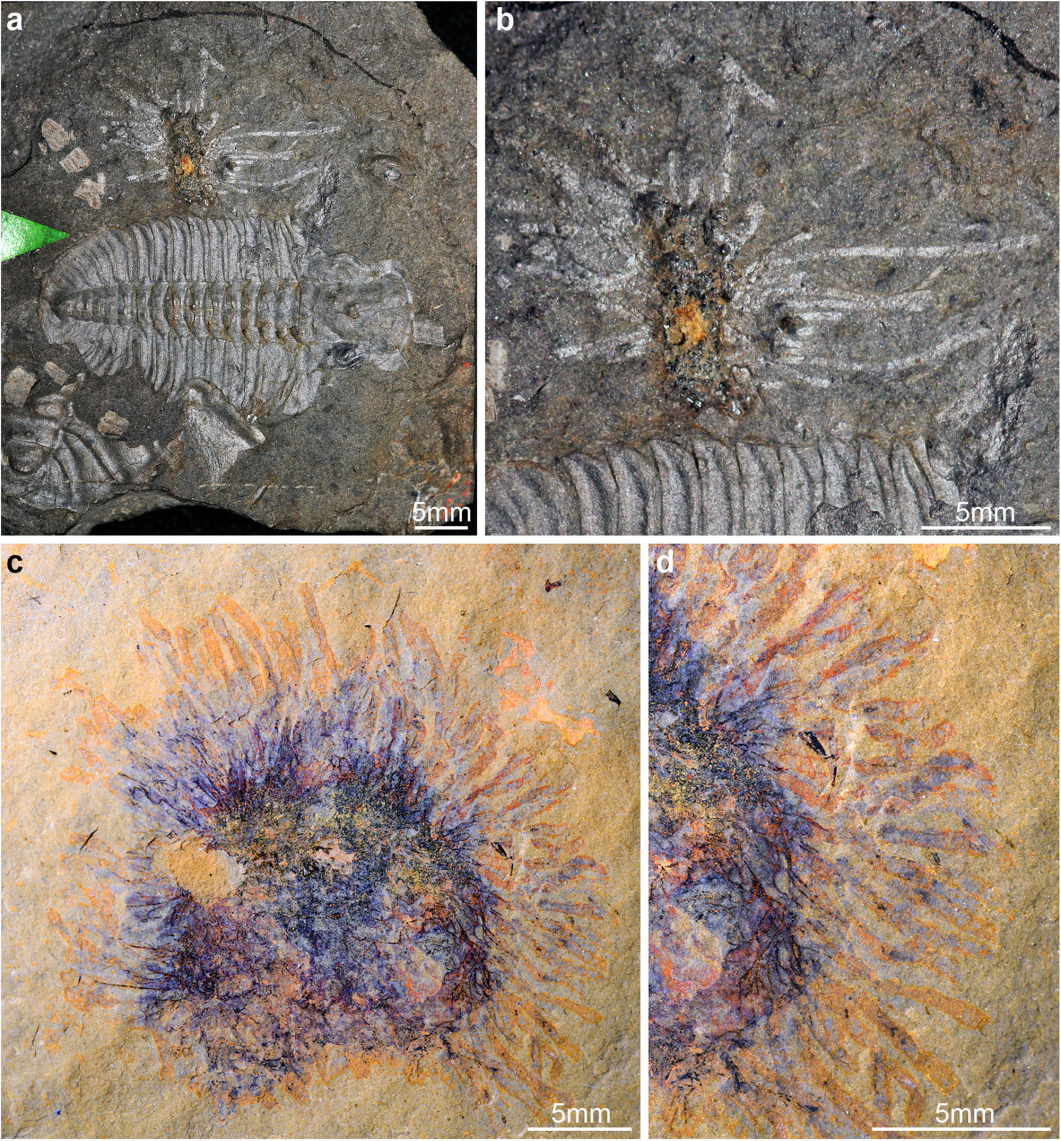
Non-dendroid benthic graptolites from Cambrian and Ordovician Burgess Shale-type biotas. **a** USNM 35406, holotype of *Yuknessia* from the mid-Cambrian Burgess Shale. **b** Close-up of *Yuknessia* showing attachment to unidentified hard substrate. **c** CAMSM X 50157, undescribed benthic graptolite from the Fezouata Shale. **d** Close-up of tubarium.

The arrangement in which the tubes appear to grow exclusively around the margins of the orthocone and not on the dorsal or ventral surfaces may also be related to the depositional environment. The colony presumably originally grew over the surface of the phragmocone indiscriminately, which begs the question of why the apparent zooid tubes appear only around the perimeter. It is possible that the burial event, being high enough energy to pass the storm-weather base as discussed above, also possessed enough energy to destroy any tubes oriented directly upward into the water column. This may have occurred in conjunction with the compression that Fezouata fossils undergo during fossilization, which would further reduce any trace of tubes not directly in line with the bedding plane. This yielded an appearance very similar to the Cambrian benthic pterobranch tube *Yuknessia*, which has also been found colonizing exclusively the perimeter of a shelly fossil assemblage^17^ (Fig. 5a, b), as well as a currently undescribed benthic pterobranch from Fezouata figured in Van Roy et al.^21^ (Fig. 5c, d).

Another possibility is that the cephalopod fell directly on top of the colony of pterobranchs, resulting in superimposition that makes it appear superficially that the tubes grow out only along the margins of the phragmocone. However, we view this possibility as unlikely for two primary reasons. First, we see no meaningful differentiation between the part and counterpart of the fossil. If the appearance of MCZ.IP.198903 resulted from the superimposition, either the part or counterpart should show some trace of the tube colony directly overlaying the phragmocone, but we do not observe this phenomenon (Fig. 2a, b; Supplementary Fig. S1). Second, MCZ.IP.198903 represents the only known specimen of a *Rhabdopleura*-like organism from the Fezouata Shale. This tally includes comprehensive studies on the diversity and local abundance of graptolites throughout the entire Fezouata Shale Formation, as well as direct examination of the ca. 6500 specimens by the present authors at the invertebrate paleontology collections of the Museum of Comparative Zoology at Harvard University. In this same census, 54 possible cephalopod phragmocone specimens were identified. While not impossible, we view it as highly improbable that the only example of this relatively small benthic graptolite colony happened to be covered by a cephalopod phragmocone by pure chance. For reference, if the probability of encountering a rhabdopleurid colony in this representative collection by pure serendipity is 0.02% (1/6500) and an orthocone is 0.8% (54/6500), the probability of both being encountered together in this collection is 0.016%. This possibility seems extremely remote unless the phragmocone acting as a substrate for the colony’s growth is actually a pre-requisite for this association to happen in the first place (see ‘The ecology and evolution of benthic graptolites’ below).

We consider another alternative interpretation, that the rhabdopleurid epibionts grew as commensals on the free-living cephalopod, as also highly unlikely. The density and maximum tube lengths expressed on the tubarium would have required its uninterrupted growth on the cephalopod for several weeks, as informed by our growth estimations, most likely disrupting its swimming mode of life and leading to its premature death. Additionally, the margins of the adjacent septa appear to be slightly irregular as opposed to perfectly parallel, as would be expected with a pristine phragmocone (see Figs. 3 and 5 from Kröger and Lefebvre^43^ for examples). This suggests some post-mortem degradation of the phragmocone before it was buried. MCZ.IP.198903 therefore provides direct evidence of epibenthic rhabdopleurid pterobranchs taking advantage of the hard substrate provided by the partially decayed orthocone in the Fezouata Shale seafloor.

MCZ.IP.198903 demonstrates that under exceptional preservation, the evidence for interspecific interactions in Early Ordovician benthic communities was similarly complex to those from the early and mid-Cambrian involving species from different phyla altogether^2,21,44^. However, the association of rhabdopleurid epibionts populating an orthocone phragmocone (*N* = 1 cephalopod host) is even rarer than exceptional interspecific interactions documented from early and mid-Cambrian Lagerstätte that involve at least one non-biomineralizing organism, such as kleptoparistic shell encrusters (*N* = 205 brachiopod hosts)^14^, brachiopods settling on mollusc spines (*N* = 2 *Wiwaxia* hosts)^15^ or vermiform symbionts (*N* = 35 tube hosts)^11^, host-specific infestations (*N* = 7 palaeoscolecid hosts)^12^, or surface foulers (*N* = 3 vetulicolian hosts)^13^. Likewise, although the Fezouata Shale features well-preserved specimens showing epibiontic interactions between conulariids and brachiopods (Fig. 1c, d), other recorded cases of possible ecological interactions in this fossil biota remain rarely documented to date (e.g., brachiopods on *Aegirocassis*; see Fig. 1b, e). We propose that the apparent discrepancies in complexity between the benthic communities of these time periods reflect taphonomic differences rather than a genuine biological signal and demonstrate the extent of ecological data that is lost without access to sites of exceptional preservation, such as the Fezouata Shale.

### Importance of rhabdopleurid graptolites in the Fezouata Shale biota

The discovery of rhabdopleurid-like pterobranch tubes in the Lower Fezouata Shale Formation has broader implications for our understanding of the graptolite paleoecology in this deposit. The Fezouata Shale biota preserves substantial graptolite biodiversity, but most species correspond to planktic ecomorphotypes^22,36^. By contrast, evidence of benthic graptolites in the Fezouata Shale biota is much more restricted in terms of their diversity and abundance, consisting mainly of the relatively rare rooted dendroids *Didymograptus* and *Dictyonema*^36,45^, as well as the problematic taxon *Webbyites* recently reinterpreted as a benthic graptolite^46^. Although the relative abundance of the different graptolite ecomorphotypes in the Fezouata Shale biota has not been comprehensively quantified, the benthic genera *Didymograptus* and *Dictyonema* make up approximately 9% (*N* = 66) of the total number of Fezouata graptolite specimens held the MCZ (*N* = 726). While further work is needed to better characterize the relative abundance of species in the Fezouata Shale, the former rough estimate demonstrates the substantial differences in graptolite frequency based on their ecology, which matches previous reports addressing their species diversity^21,36^. Van Roy et al.^21^ figured the only putative graptolite from the Fezouata Shale biota that is comparable with MCZ.IP.198903; the specimen (CAMSM X 50157; deposited at the Sedgwick Museum at the University of Cambridge) consists of a dense bush-like tubarium with at least 60 individually identifiable tubes that radiate from a common area, presumably at their base (Fig. 5c, d). Details of the fine morphology of the tubes remain undescribed, but the growth pattern of the tubarium strongly suggests that the specimen also represents a benthic graptolite colony that likely grew attached to a hard substrate, although its precise affinities are less well resolved compared to MCZ.IP.198903. Taken together, these two specimens and the recently redescribed *Webbyites*^46^ reflect the totality of the non-dendroid benthic graptolite species reported from the Fezouata Shale biota to date, which is striking considering the substantial diversity and abundance of benthic organisms known from this exceptional fossil deposit including over a hundred species and thousands of individuals^22–33^.

### The ecology and evolution of benthic graptolites

Planktic graptolites were extremely successful for tens of millions of years, to the extent that they are one of the most important biostratigraphic taxa in the beds where they occur^36,37,45,47^. However, the heyday of planktic graptolite diversity was over by the late Ordovician^47,48^, and the group was entirely extinct by the mid-Carboniferous^45^. Benthic graptolite diversity has been less spectacular by contrast but much more stable over a greater timeframe. The fossil record of benthic graptolites extends at least as far back as the middle Cambrian^49^ and possibly from the Fortunian^50^. Despite leaving a more modest mark in the geologic record compared to their planktic counterparts, benthic pterobranchs still exist half a billion years later, as embodied by *Rhabdopleura*^37^. In this context, the rhabdopleurid-like tubes of MCZ.IP.198903 provide a deeper insight into these highly divergent eco-evolutionary dynamics. Planktic graptolites diversified as part of the Ordovician planktonic revolution, which entailed a greater proportion of overall energy in marine systems moving into the water column^51,52^. Benthic graptolite colonies begin with individual larvae which settle, metamorphose, and build elaborate tubaria in a series of increasingly complex stages^37,53,54^. We hypothesize that the flocculent, loose fine-grained sediment that predominated Cambro-Ordovician Lagerstätte might have been detrimental to the growth and proliferation of benthic pterobranchs, whereas the water column was becoming more conducive to the ecology of planktic forms. This hypothesis also finds support in the wide variety of early hemichordates found among Cambrian Lagerstätte, which, while retaining the body plan of the enteropneusts, have a pterobranch-like ecology^55,56^. It seems like that while flocculent sediments were not conducive to pterobranch larval establishment, tube-building as a behavior was still viable among macroscopic, direct-developing hemichordates with analogous sessile and filter-feeding modes of life. This is also partially borne out by the graptolite diversity of the Fezouata Shale biota, which is overwhelmingly dominated by planktic representatives^21,36^. The dense aggregations of benthic pterobranchs exemplified by MCZ.IP.198903, and possibly also by CAMSM X 50157^21^ (Fig. 5c), likely reflect the high specificity of their ecological requirements for initial settlement, metamorphosis, and post-larval growth. While most local environments were not ideal for these taxa, those that were favorable became heavily populated, to the extent that even recalcitrant corpses represented critical islands of habitability for these organisms. While benthic graptolites like those we have described in this study may have been proportionally rarer than their planktic counterparts, they are ultimately the forms that have endured and survived as part of the modern marine biosphere. It should also be re-emphasized that despite benthic pterobranchs being a minor, if not entirely absent, component of the most well-studied Cambrian Lagerstätten^2^, they actually precede the dominance of later planktic forms. Examples include the fragmentary *Sokoloviina* that has been suggested as a partial pterobranch tube and dates back to the Fortunian^50^, as well as a variety of dendrograptids from the Furongian-aged Guole biota^57^. These taxa and other benthic graptolites are not typically found with direct evidence of attachment, and thus the exact extent to which they relied on highly specific substrate types is debatable. However, at least one possible specimen of Rhabdopleura from Guole has been found attached to the tube-shaped fossil *Sphenothallus*^57^. Clearly, the relative rarity of hard compared to soft substrates did not preclude pterobranch diversification but may have acted as an ecological limitation to the rapid speciation that occurred once they were able to exploit the planktonic realm.

To that end, this new fossil association also underscores the post-mortem role of taxa in perpetuating diversity. In this case, a component of a cephalopod’s anatomy has been used as a substrate for the growth of dozens of colonial individuals in the only instance of this taxa discovered among thousands of fossils excavated^26^. This type of opportunism is known among pterobranchs not only from the Ordovician but in the holotype of the middle Cambrian *Yuknessia*^17^, as well as modern *Rhabdopleura*, which colonize the dead shells of bivalves^58^. The latter example is particularly striking in this context, demonstrating that hemichordates have been making opportunistic use of mollusc shells as hard substrates for nearly 480 million years.

## Methods

### Locality and acquisition information

The studied specimen (MCZ.IP.198903; Figs. 1a and 2a–d) is housed in the Invertebrate Paleontology collections at the Museum of Comparative Zoology at Harvard University. The specimen is part of a larger collection produced by Mohammed ‘Ou Said’ Ben Moula from the Draa Valley in the Zagora region of Morocco, purchased in 2019 from Lahcen Ben Moula (Address in Morocco: Ksar Taichouta, Alnif, Tinghuir), and exported by Brahim Tahiri (108 Cite Es-salem, Erfoud, Morocco) with the approval of the Ministry of Mines in Rabat (Invoice N. 92/E/21). The fossil pit (w3w code: engenders.downwards.reanimate; coordinates N 30°29.690’ W 5°50.827’) that produced MCZ.IP.198903 also contains the planktic graptolites *Seganograptus murrayi* and *Paradelograptus norvegicus*, which indicates a late Tremadocian age^36^, and a stratigraphic provenance from the Lower Fezouata Formation.

### Light photography and micro-CT imaging

MCZ.IP.198903 was photographed under cross-polarized light using a Nikon D850 DSLR camera fitted with a Macro Nikkor 60 mm lens and a Zeiss Axiocam fitted to a Zeiss Stemi 305 stereo microscope. MCZ.IP.198903 was CT scanned using the X-tek HMXST 225 micro-CT x-ray imaging machine at the Harvard Center for Nanoscale Systems. The part (smaller slab) was scanned with a 1 mm aluminum filter with energy set at 95 kV/120 μA and an imaging pixel size of 33.38 μm. The counterpart (larger slab) was scanned with a 2.5 mm copper filter with energy set at 203 kV/227 μA and an imaging pixel size of 44.78 μm. The scans were reconstructed in Nikon’s CT Pro 3D software and visualized in Dragonfly 2020.2 [Computer software]. Object Research Systems (ORS) Inc, Montreal, Canada, 2020; software available at http://www.theobjects.com/dragonfly.

### Reporting summary

Further information on research design is available in the Nature Portfolio Reporting Summary linked to this article.

### Supplementary information

Supplementary Information

Reporting Summary

## Data Availability

All data are available within the main manuscript and the supplementary information of this paper. Figured fossil specimens are housed at the Invertebrate Paleontology Collection at the Museum of Comparative Zoology, Harvard University (MCZ.IP), and the Invertebrate Paleontology Collection at the Peabody Museum of Natural History, Yale University (YPM.IP).
