# Supplementary figures and images for: Rhabdopleurid epibionts from the Ordovician Fezouata Shale biota and the longevity of cross-phylum interactions
Source: Commun Biol. 2023 Oct 11;6:1002. doi: 10.1038/s42003-023-05377-x (PMC10567727; doi:10.1038/s42003-023-05377-x)

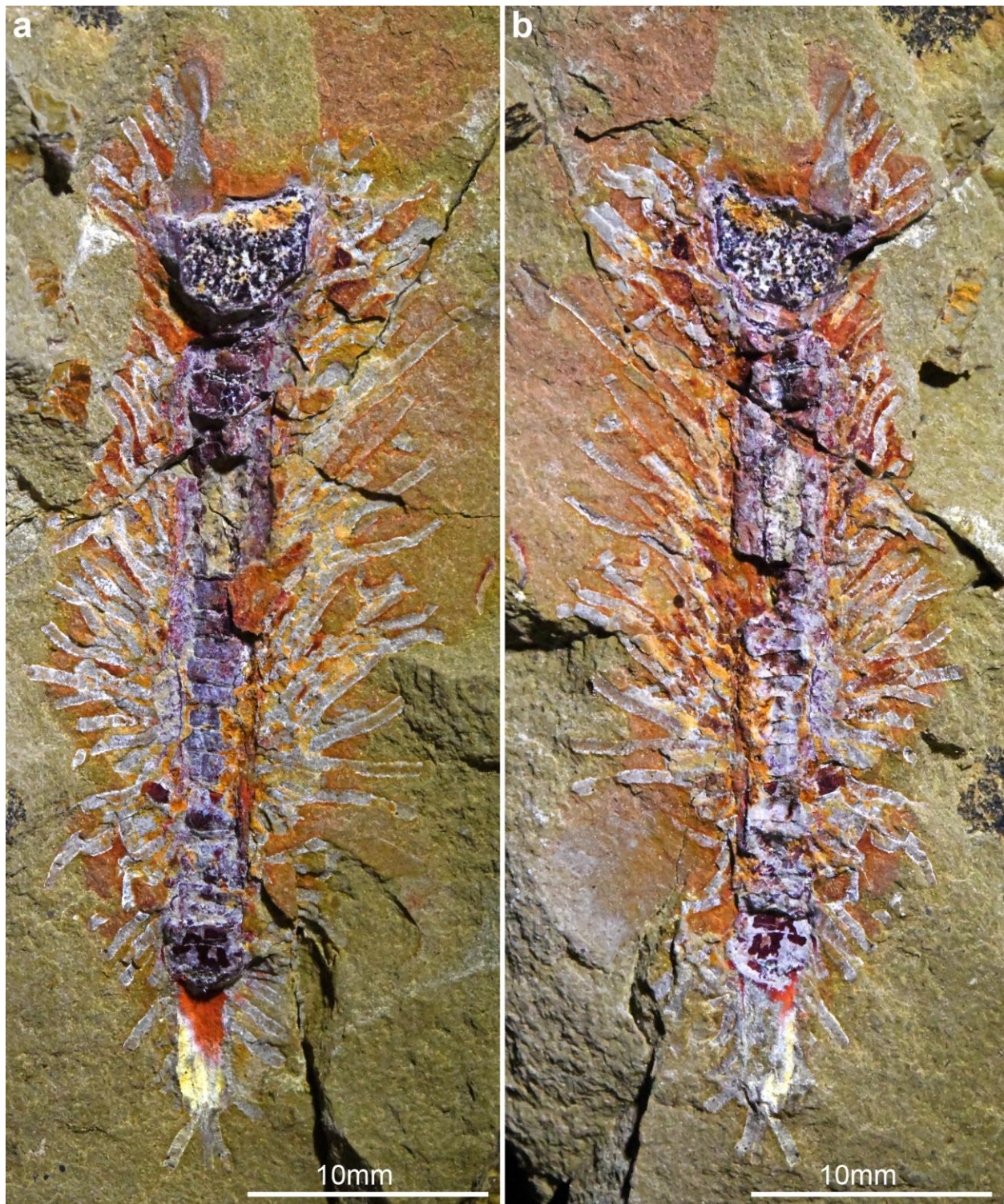

**Figure S1.** High-resolution digital photographs of MCZ.IP.198903. **a**, Part. **b**, Counterpart.

Supplement: Supplementary file 1 — Supplementary Information [file 42003_2023_5377_MOESM1_ESM.pdf]
